# The p53-dependent apoptotic pathway of breast cancer cells (BC-M1) induced by the bis-type bioreductive compound aziridinylnaphthoquinone

**DOI:** 10.1186/bcr939

**Published:** 2004-11-04

**Authors:** Yu-Ping Yang, Hsien-Shou Kuo, Hsin-Da Tsai, Yi-Chen Peng, Yuh-Ling Lin

**Affiliations:** 1 Department of Biochemistry, College of Medicine, Taipei Medical University, Taiwan, ROC; 2 Department of Medicine, College of Medicine, Fu-Jen Catholic University, Taipei Hsien, Taiwan, ROC

**Keywords:** apoptosis, bioreductive compound, bis-type aziridinylnaphthoquinone, breast cancer cells (BC-M1 and MCF-7)

## Abstract

**Introduction:**

Several aziridinylbenzoquinone drugs have undergone clinical trials as potential antitumor drugs. These bioreductive compounds are designed to kill cells preferentially within the hypoxia tumor microenvironment. The bioreductive compound of bis-type naphthoquinone synthesized in our laboratory, 2-aziridin-1-yl-3-[(2-{2-[(3-aziridin-1-yl-1,4-dioxo-1,4-dihydronaphthalen-2-yl)thio]ethoxy}ethyl)thio]naphthoquinone (AZ-1), had the most potent death effect on the breast cancer cells BC-M1 in our previous screening. In the present study, we determined that the mechanism of the death effect of BC-M1 cells induced by AZ-1 was mediated by the apoptosis pathway.

**Methods:**

We evaluated the cytotoxicity of AZ-1 and the anti-breast cancer drugs tamoxifen and paclitaxel to BC-M1 cells and MCF-7 cells by the MTT assay and measured the apoptosis phenomena by Hoechst 33258 staining for apoptotic bodies. We also quantified the sub-G_1 _peak area and the ratio of the CH_2_/CH_3 _peak area of the cell membrane in BC-M1 cells by flow cytometry and ^1^H-NMR spectra, respectively.

The apoptosis-related protein expressions, including p53, p21, the RNA-relating protein T-cell restricted intracellular antigen-related protein, cyclin-dependent kinase 2 (cell cycle regulating kinase) and pro-caspase 3, were detected by western blot, and the caspase-3 enzyme activity was also quantified by an assay kit.

**Results:**

AZ-1 induced two of the breast cancer cell lines, with IC_50 _= 0.51 μM in BC-M1 cells and with IC_50_= 0.57 μM in MCF-7 cells, and showed less cytotoxicity to normal fibroblast cells (skin fibroblasts) with IC_50_= 5.6 μM. There was a 10-fold difference between two breast cancer cell lines and normal fibroblasts. Of the two anti-breast cancer drugs, tamoxifen showed IC_50_= 0.12 μM to BC-M1 cells and paclitaxel had much less sensitivity than AZ-1. The expression of p53 protein increased from 0.5 to 1.0 μM AZ-1 and decreased at 2.0 μM AZ-1. The p21 protein increased from 0.5 μM AZ-1, with the highest at 2 μM AZ-1. Regarding the AZ-1 compound-induced BC-M1 cells mediating the apoptosis pathway, the apoptotic body formation, the sub-G_1 _peak area, the ratio of CH_2_/CH_3 _of phospholipids in the cell membrane and the enzyme activity of caspase-3 were all in direct proportion with the dose-dependent increase of the concentration of AZ-1. The death effect-related proteins, including T-cell restricted intracellular antigen-related protein, cyclin-dependent kinase 2, and pro-caspase-3, all dose-dependently decreased with AZ-1 concentration.

**Conclusions:**

The AZ-1-induced cell death of BC-M1 cells mediating the apoptosis pathway might be associated with p53 protein expression, and AZ-1 could have the chance to be a candidate drug for anti-breast cancer following more experimental evidence, such as animal models.

## Introduction

The bioreductive drugs, aziridinylbenzoquinones, are a class of compounds designed to exploit one of the features of solid tumor biology caused by an inadequate blood supply to the solid tumor; namely, tumor hypoxia. Such regions generally are resistant to radiation and other oxygen-requiring treatment [1-4]. The ideal bioreductive drug should be administered as an inactive prodrug that is only activated under low-oxygen conditions by one-electron or two-electron reductase [5]. The aziridine-substituted benzoquinones such as mitomycin C, RH1, and E09 are three principal aziridinylquinone-class hypoxia-specific cytotoxins that are being developed for clinical use [6-8]. These agents are composed of an aziridinyl moiety on a quinone structure, and they convert on reductive metabolism to a bifunctional alkylating species that can cross-link DNA in the major grove that interacts predominantly on guanine-N7 [9]. These agents probably produce their major cytotoxic activities through the formation of DNA cross-links.

Tumor tissue with lower oxidative reduction (redox) potential relative to most normal tissue could increase reductive activation of these quinone derivatives in the tumor [2]. The selectivity of bioreductive drugs is therefore governed not only by the difference in oxygen tension between the tumor and normal tissue, but also by levels of enzymes catalyzing bioreductive activation such as DT-diaphorase [4,10,11]. This fact led to the publication in 1990 of the concept of 'enzyme-directed bioreductive development' by Workman and Walton [12].

In many cases, the biological activity of quinone is attributed to the ability of accepted electrons to form the corresponding radical anion or dianion species. A quinone moiety substituted with aziridine has been shown to be a potent alkylating agent as a result of bioreduction by the one-electron reducing enzymes (e.g. NADPH cytochrome P450 reductase, cytochrome b5 reductase) or by two-electron reducing enzymes ([NADP]H oxidoreductase, NQO1) to form the corresponding aziridinyl hydroquinone [13-15]. The hydroquinone moiety in the corresponding aziridinyl hydroquinone effectively changes the p*K *value of the aziridine ring such that it is protonated and becomes activated toward nucleophilic attack under physiological pH.

In previous work in our laboratory, we biosynthesized the bis-type of bioreductive aziridinylnaphthoquinone series compounds and evaluated their anti-cancer activity [16,17]. In the case of di-aziridinyl-substituted quinone, this highly cytotoxic bifunctional alkylating agent can cross-link DNA in cells that results in induction of a complex cellular mechanism leading to cell death by apoptosis or necrosis [18]. Based on our previous results, the compound 2-aziridin-1-yl-3-[(2-{2-[(3-aziridin-1-yl-1,4-dioxo-1,4-dihydronaphthalen-2-yl)thio]ethoxy}ethyl)thio]naphthoquinone (AZ-1) had the most potent death effect on breast cancer cells (BC-M1) and less cytotoxicity to normal fibroblast cells (skin fibroblasts). In this study, we were interested in determining the cytotoxicity of AZ-1 to our localized cell line (BC-M1 cells) and to determine the mechanism by which this occurs. We compared the death effect of two cell lines (BC-M1 and the estrogen-receptor-positive cell line MCF-7) induced by AZ-1, and also compared the cytotoxicity of AZ-1 with two clinical anti-breast cancer therapies, tamoxifen (aromatase inhibitors) and paclitaxel, in the cell viability of BC-M1 cells.

## Materials and methods

### Materials

RPMI 1640 medium, DMEM medium, fetal bovine serum, 2 mM L-glutamine, MEM non-essential amino acid, trypsin-EDTA solution, PBS, Hank's balanced salt solution, pencillin-streptomycine and fungizone were purchased from Gibco Laboratories (Grand Island, NY, USA). NaHCO_3_^-^, MTT, trypan blue, EDTA, propidium iodide, Hoechst 33258, paclitaxel and tamoxifen were purchased from Sigma Chemical Co. (St Louis, MO, USA). The primary antibodies of T-cell restricted intracellular antigen-related protein (TIAR), pro-caspase and p53, cyclin-dependent kinase (cdk) 2 and cyclin B were purchased from BD Transduction Laboratories (BD Bioscience, Palo Alto, CA, USA). Peroxidase-conjugated affinity-purified goat anti-mouse IgG was purchased from Jackson Immuno Research Laboratories (West Grove, PA, USA). The chamber slide was purchased from NUNC (Roskilde, Denmark). All other chemicals were purchased from Merck (Darmstadt, Germany). AZ-1 was obtained from total synthesis in our laboratory, was dissolved in dimethsulfoxide before experimental use and was aliquoted to be stored at -20°C, with stability for several years.

### Methods

#### Human cell lines cultured

The BC-M1 (human breast adenocarcinoma) cell line was cultured in RPMI 1640 medium with 10% fetal bovine serum, 2 mM L-glutamine, and 25 mM Hepes. Skin fibroblasts and MCF-7 cells were cultured in DMEM medium with 10% fetal bovine serum, 2 mM L-glutamine, and MEM nonessential amino acid. The cell culture media for the two cell lines all contained pencillin-streptomycine and fungizone. All cells were incubated in a humidified atmosphere of 5% CO_2 _at 37°C. Cell cultures were subcultured once or twice weekly using trypsin-EDTA to detach the cell from their culture flask. The numbers of cells were counted after trypsinization by a Neubauer hemocytometer (VWR Scientific Corp., Philadelphia, PA, USA).

#### Cytotoxicity determined by MTT for cell viability

The MTT assay was performed according to the method of Skehan and colleagues [19]. One day before drug application, cells were seeded in 96-well flat-bottomed microtiter plates (3000–5000 cells/well). Cells were incubated for 24 hours with various drugs, and applied as serial dilutions (100 μl/well) at various concentrations. Twenty microliters of MTT (5 mg/ml) were added to each well and incubated for 4 hours at 37°C. The formazan product was dissolved by adding 100 μl dimethylsulfoxide to each well, and the plates were read at 550 nm. All measurements were performed in triplicate and each experiment was repeated at least three times. The IC_50 _value was calculated from the 50% formazan formation compared with a control without addition of drugs.

#### Apoptoic body stained by Hoechst 33258

The cells were cultured in RPMI 1640 complete medium for BC-M1 cells and in DMEM complete medium for MCF-7 cells on a chamber slide (1 × 10^4 ^cells/ml). Various concentrations of AZ-1 compound were added and incubated at 37°C. After 24 hours of incubation, the cultured medium was removed and the cells were fixed by acetic acid/methanol (1:3) solution for 10 min. In the following step, the fixed solution was removed and cells were air-dried for another 10 min. The cell was stained by Hoechst 33258 stain solution (0.5 μg/ml in Hank's balanced salt solution) at room temperature for 30 min. After staining, the solution was removed, the cell was washed three times with distilled water and then one drop of mounting solution (0.1 M citric acid:0.2 M disodium phosphate:glycerol, 1:1:2) was added before being covered by a cover slide. Apoptotic cells showed blue, peripherally clumped or fragmented chromatin.

#### Apoptosis analysis by flow cytometry

The apoptotic nuclei of BC-M1 cells induced by AZ-1 were also identified by the flow cytometry analysis method as described by Dive and colleagues with minor modification [20]. The BC-M1 cells were treated with various concentrations of AZ-1 for 24 hours. Cells were harvested and DNA was stained with propidium iodide. The DNA content was measured by flow cytometry (Becton Dickinson FACScan, San Jose, CA, USA).

#### Western blot analysis

This analysis method was that according to Bacus and colleagues with slight modifications [21]. Briefly, cells were collected from a 100 mm cultured dish after challenge by various concentrations of AZ-1 compound at 24 hours. Cell pellets were spun-down by centrifugation (1000 × *g *× 20 min). Pellets were resuspended in cold buffer (10 mM HEPES [pH 7.9], 1.5 mM MgCl_2_, 10 mM KCl, 0.5 mM dithiothreitol, 0.5 mM phenylmethylsulfonyl fluoride, 1 mM benzamidine, 30 mg/ml leupeptin, 5 mg/ml aprotinin, and 5 mg/ml pepstatin A; all from Sigma) and were incubated on ice for 5 min and lysed by sonication. Cell lysate (25 μg) was separated by 12% SDS-PAGE and transferred onto polyvinylidene difluoride membranes (Amersham, Little Chalfont, UK). Blots were incubated with blocking buffer (11 mM Tris-vase [pH 7.4], 154 mM NaCl, and 5% skim milk), washed by washing buffer (11 mM Tris-vase [pH 7.4], 154 mM NaCl, and 0.1% Tween-20) and incubated with specific antibodies to probe specific proteins. The primary antibodies were from mouse anti-human monoclonal antibodies (Imgenex Co., San Diego, CA, USA). The secondary antibody (Jackson ImmunoResearch Laboratories) was conjugated with horseradish peroxidase at an appropriate dilution by blocking buffer. The dilution factors for the primary and secondary antibodies were various (dependent on different proteins) and 1:5000, respectively. The primary antibodies used were a mouse monoclonal antibody to GAPDH and β-actin with dilution factors 1:2000 (Biogenesis, Poole, UK) and 1:10,000 (Calbiochem, San Diego, CA, USA), respectively. Immunodetection was carried out using enhanced chemiluminescence (NEN, Boston, MA, USA) detection system. Quantification of various protein expressions were achieved by measuring the intensity of chemiluminescence of the second antibody using a densitometer (BioRad Gel Doc 2000 software, analyzed using Gel Doc; BioRad Laboratories, Hercules, CA, USA) The values in the relative expression-quantifying table of various proteins represent the relative amounts of protein expression with respect to GAPDH or β-actin expression divided by its control.

#### Caspase-3 activity assay

The BC-M1 cells were treated with various concentrations of AZ-1 for 24 hours. The cells were harvested and washed with PBS buffer twice. The caspase-3 activity of BC-M1 cells challenged by AZ-1 was measured by the CPP32/caspase-3 protease colorimetric assay kit, with the assay method according to the procedure described by the manufacturer (Chemicon, Temecula, CA, USA). Briefly, the cells were first lysed by cold lysis buffer (supplied by the assay kit) for 10 min. The cell lysate was then centrifuged to remove the cell debris, and the protein concentration determined by Barford reagent (BioRad Laboratories). The supernatant was ready to detect the caspase-3 activity.

#### Apoptosis analysis by ^1^H-NMR spectra

^1^H-NMR spectroscopy was performed *in vitro *using methods previously described by Francis and colleagues with minor modification [22]. Briefly, 5 × 10^7 ^cells were harvested and washed twice with PBS medium made with D_2_O (90% purity), suspended in a final volume of 500 μl, and were placed immediately on ice until data acquisition. Samples were analyzed on a 400 MHz high-resolution Brucker CSI Omega spectrometer (Brucker, Karlsruhe, Germany) at 18°C, with pulse acquisition, a 90° flip angle, a repetition time of 10 s, 64 or 128 excitations (depending on the desired signal-to-noise ratio), 8 k points, and a 5 kHz bandwidth. A coaxial tube filled with trimethylsialoproponic acid, 0.1% solution in D_2_O, was used as reference (0.0 ppm) for each experiment. The relative areas underneath the CH_2 _and CH_3 _resonance curves (at 1.3 ppm and 0.9 ppm, respectively) were calculated by integration of the proton spectrum using the trough between the CH_2 _and CH_3 _resonances as a baseline reference.

## Results

### The cytoxicity of AZ-1 to BC-M1 cells and MCF-7 cells

**Figure 1:**
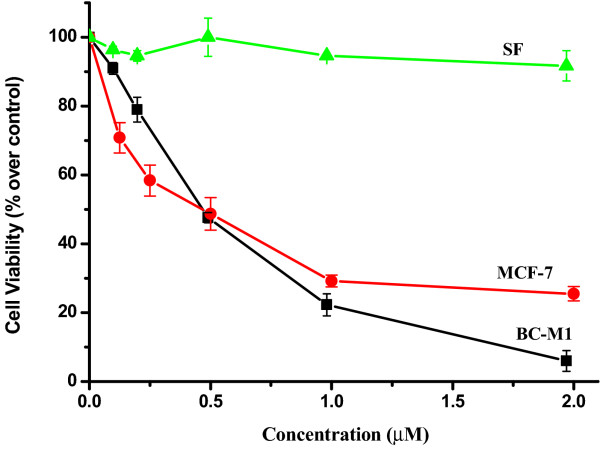
Bis-aziridinylnapthoquinone (AZ-1) inhibited the proliferation of three cell lines, skin fibroblasts (SF), BC-M1 and MCF-7. Cells were seeded for 18 hours before the addition of AZ-1 with various concentrations. The death effects of these three cell lines induced by various concentrations of AZ-1 were compared by the MTT assay. The MTT assay was used to determine the cell viability after an additional 24 hours of culture. Data are from quadruplet wells and are representative of three separate experiments.

**Figure 2:**
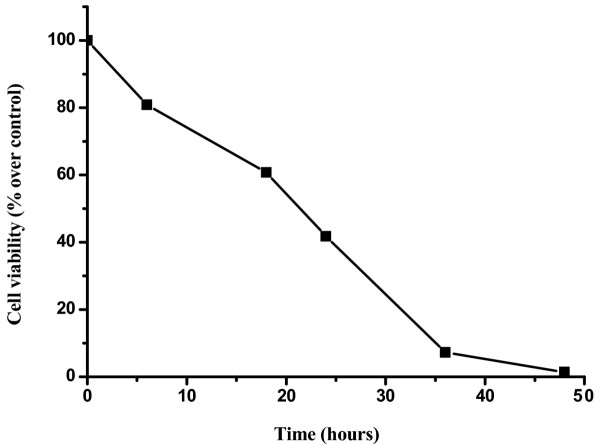
Bis-aziridinylnapthoquinone (AZ-1) inhibited the proliferation of cell line BC-M1. Shown is the cell viability of BC-M1 cells at various times using the IC_50 _dose (0.51 μM) of AZ-1 challenge. The MTT assay was used to determine the cell viability. Data are from quadruplet wells and are representative of three separate experiments.

**Figure 3:**
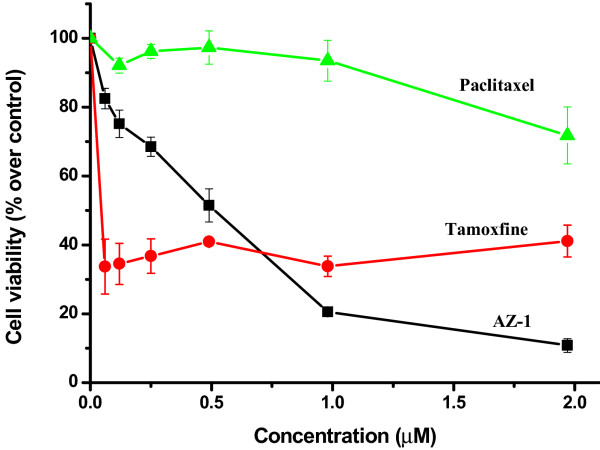
Comparison of the death effect of BC-M1 cells induced by various concentrations of bis-aziridinylnapthoquinone (AZ-1), paclitaxel and tamoxifen by the MTT assay. The MTT assay was used to determine the cell viability after an additional 24 hours of culture. Data are from quadruplet wells and are representative of three separate experiments.

The cytotoxicities of the IC_50 _value in AZ-1 to BC-M1 cells and MCF-7 cells were 0.51 μM and 0.57 μM in a dose-dependent manner, respectively. The response of these two cell lines (MCF-7 and BC-M1) to AZ-1 was very similar to cell viability in a dose-dependent manner. In the normal fibroblast cell (skin fibroblast) there was still a 90% survival rate at 2 μM (Fig. 1). According to the time-dependent treatment of AZ-1 using an IC_50 _concentration of 0.51 μM to BC-M1 cells, the cell viability was less than 10% after 36 hours of treatment in a time-dependent manner (Fig. 2). The two clinical therapies paclitaxel and taxmoxifen were compared regarding cytoxicity in BC-M1 cells with AZ-1, and the estrogen receptor antagonist taxmoxifen and AZ-1 were more potent than paclitaxel. The taxmoxifen had a low IC_50 _value of 0.05 μM, which is lower than AZ-1, with a plateau concentration from 0.05 μM to 2 μM (Fig. 3).

### Apoptosis assay by flow cytometry and Hoechst staining

**Figure 4:**
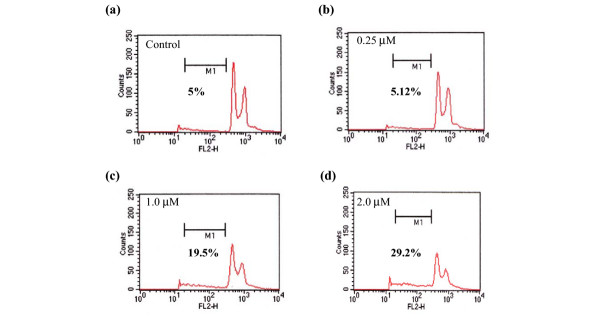
Apoptosis induced by bis-aziridinylnapthoquinone (AZ-1). BC-M1 cells were treated in 24-hour culture with **(a) **dimethylsulfoxide control, **(b) **0.2 μM AZ-1, **(c) **1.0 μM AZ-1, or **(d) **2.0 μM AZ-1 before propidium iodide staining and analysis of the DNA content. Apoptosis is apparent from the large population of cells with increased DNA content in the sub-G_1 _area (M1 area). FL2-H, fluorescence two-peak high.

**Figure 5:**
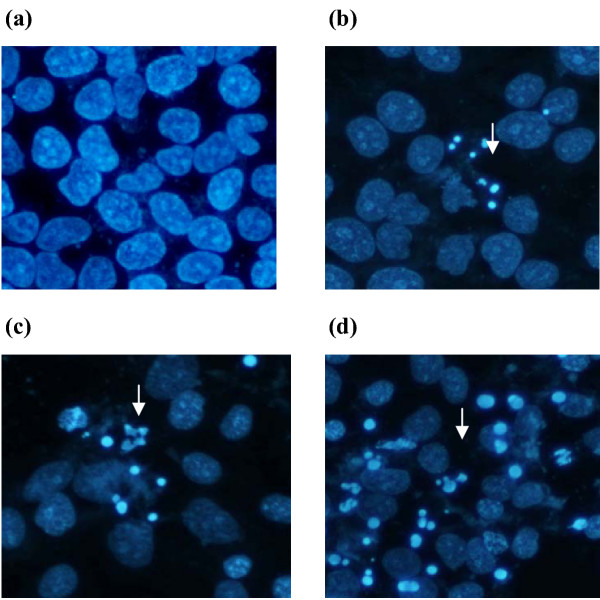
Apoptosis induced by bis-aziridinylnapthoquinone (AZ-1) in BC-M1 cells. The BC-M1 cells were treated in 24-hour culture with **(a) **dimethylsulfoxide control, **(b) **0.2 μM AZ-1, **(c) **1.0 μM AZ-1, or **(d) **2.0 μM AZ-1 before Hoechst staining and analysis of the DNA nuclei. Apoptotic cells showed as blue, peripherally clumped or fragmented chromatin as indicated by the arrows.

**Figure 6:**
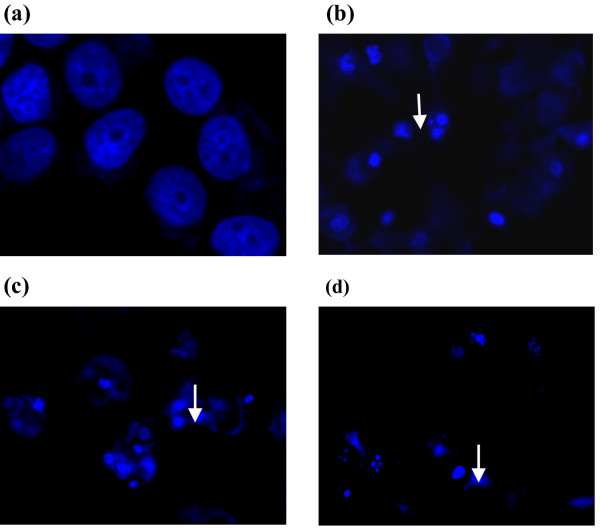
Apoptosis induced by bis-aziridinylnapthoquinone (AZ-1) in MCF-7 cells. The MCF-7 cells were treated in 24-hour culture with **(a) **dimethylsulfoxide control, **(b) **0.2 μM AZ-1, **(c) **1.0 μM AZ-1, or **(d) **2.0 μM AZ-1 before Hoechst staining and analysis of the DNA nuclei. Apoptotic cells showed blue, peripherally clumped or fragmented chromatin as indicated by the arrows.

Analysis of the DNA content of cells was used to determine whether cell apoptosis was induced by AZ-1. The data of the sub-G_1 _area indicated that BC-M1 cells had a significant population of cell apoptosis in the sub-G_1 _area on treatment with AZ-1 for 24 hours compared with dimethylsulfoxide alone (Fig. 4). The sub-G_1 _area started at 1.0 μM AZ-1 and exhibited a large increase in apoptotic cells identified as subcellular populations with decreasing DNA. The BC-M1 cells treated with 2.0 μM AZ-1 exhibited the largest apoptotic area (29.2%). This is similar to the apoptotic bodies observed using the Hoechst stain (Fig. 5). The Hoechst staining method was used to identify the apoptotic nuclei in BC-M1 cells and MCF-7 cells. Apoptotic cells that contained the apoptotic bodies showed blue peripherally clumped or fragmented chromatin, as indicated by arrows in Figs 5 and 6. From the visual observation of the Hoechst staining results, the BC-M1 cells treated with 2.0 μM had the highest number of apoptotic bodies (Fig. 5). The formation of apoptotic bodies was observed obviously at concentrations as low as 0.5 μM AZ-1 in MCF-7 cells (Fig. 6).

### Tumor cell apoptosis associated with protein expression and caspase-3 activity

**Figure 7:**
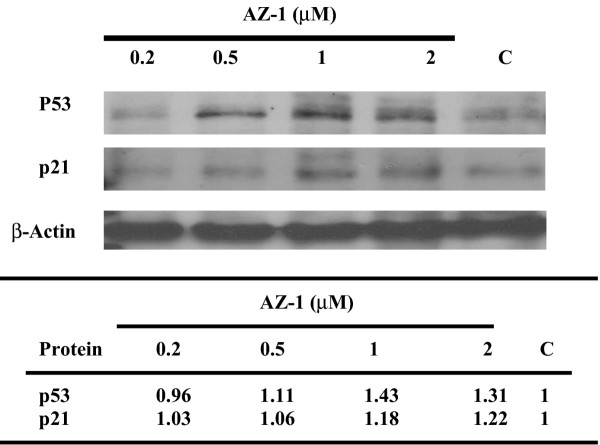
Bis-aziridinylnapthoquinone (AZ-1) alters the expression of apoptotic proteins p53 and p21. The protein expression of p53 and p21 were assessed by immunoblot. The BC-M1 cells were treated with various concentration of AZ-1 (lanes 1–4: 0.2 μM, 0.5 μM, 1.0 μM, and 2.0 μM) for 24 hours and cell lysate was prepared for analysis. Lane 5, untreated control (C). β-actin was an internal control on BC-M1 cells.

**Figure 8:**
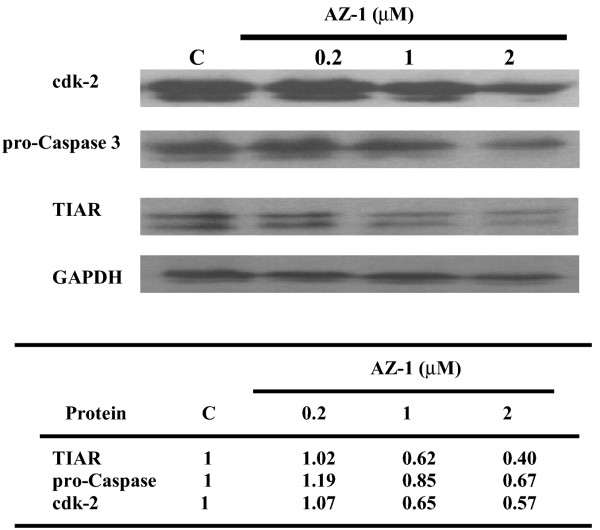
Bis-aziridinylnapthoquinone (AZ-1) alters the expression of apoptotic proteins. The protein expression of T-cell restricted intracellular antigen-related protein (TIAR), pro-caspase 3 and cyclin-dependent kinase (cdk2) were assessed by immunoblot. The BC-M1 cells were treated with various concentration of AZ-1 (lanes 2–4: 0.2 μM, 1.0 μM, and 2.0 μM) for 24 hours and cell lysate was prepared for analysis. Lane 1, untreated control (C). GADPH was an interna l control on BC-M1 cells.

**Figure 9:**
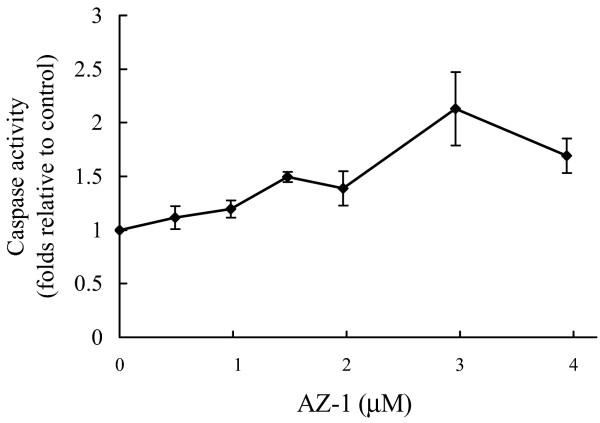
The enzyme activity of caspase 3 in BC-M1 cells was induced by bis-aziridinylnapthoquinone (AZ-1). Cells were seeded for 24 hours before the addition of AZ-1 in various concentrations. The cell lysate was prepared for analysis by a CPP32/caspase 3 colorimetric assay kit according to the manufacturer's instructions. The enzyme activity is shown as the fold increase relative to the control.

We next set out to determine whether the induction of BC-M1 cell apoptosis by AZ-1 was associated with expression of apoptosis-related proteins. We found that AZ-1 induced changes in BC-M1 cell expression of the checkpoint protein p53 and the cell arrest protein p21 in a dose-related manner (Fig. 7). The p53 protein showed increasing expression from 11% to 43% at 0.5 μM and 1 μM AZ-1, and then decreased to 31% at the 2 μM concentration of AZ-1, and p21 protein increased from 6% to 22% from the 0.5 μM to 2 μM concentrations of AZ-1 added to BC-M1 cells for 24 hours compared with control, respectively. The other proteins including TIAR, pro-caspase protein and cell cdk2 also showed a dose-dependent decreasing manner (Fig. 8). From the western blot results and the values in the relative protein expression-quantifying table (Figs 7 and 8) it was revealed that the expression of proteins was affected by various concentrations of AZ-1 in BC-M1 cells after 24 hours of treatment, and these relative protein expressions were compared with the control. The cdk2, pro-caspase protein and TIAR were reduced to about 62%, 85% and 65%, and to 40%, 67% and 57% when BC-M1 cells treated with 1 μM AZ-1 and 2 μM AZ-1 for 24 hours compared with control, respectively. Based on the results of western blot analysis in the expression of pro-caspase protein, we determined the enzyme activity of caspase-3 in BC-M1 cells after challenge by various concentrations of AZ-1 from 0.5 μM to 4 μM for 24 hours. The enzyme activity of caspase-3 dose-dependently increased with concentrations of AZ-1. The activity was more than twofold over the control in BC-M1 cells after 3 μM AZ-1 treatment for 24 hours (Fig. 9).

### Assay of the apoptosis signal by ^1^H-NMR

**Figure 10:**
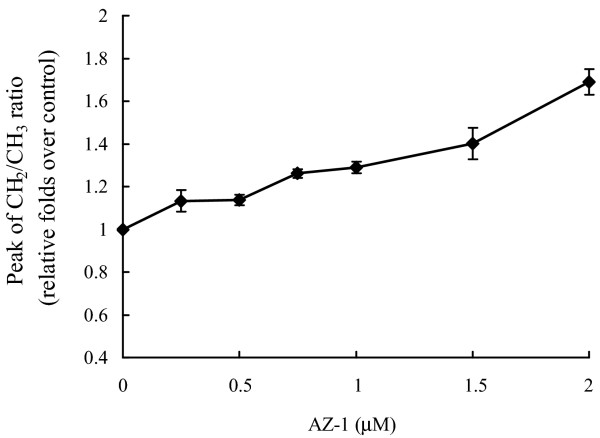
Plot of the ratio of CH_2_/CH_3 _peak area according to NMR spectra as the dose of bis-aziridinylnapthoquinone (AZ-1)-treated BC-M1 cells. Cells were seeded for 24 hours before the addition of AZ-1 in various concentrations. The ratio of CH_2_/CH_3 _signal intensity was plotted against the various concentrations of AZ-1.

According to some previous reports, the ratio of the CH_2 _and CH_3 _peak area on the cell membrane was directly in proportion with the signal of apoptosis. From our results we also observed the same phenomena that the ratio of the CH_2 _and CH_3 _peak area was increasing according to the concentration of AZ-1 (Fig. 10). It was about 1.7-fold higher than the control at 2 μM AZ-1 treatment in BC-M1 cells.

## Discussion

Breast cancer is the most common malignancy in women, and it is highly curable if diagnosed at early stage. It is now well established that adjuvant systemic therapy improves survival in patients with early-stage breast cancer [23,24]. Treatment options for early-stage breast cancer include chemotherapy (e.g. anthrocyclines, taxanes) and hormone therapy (e.g. tamoxifen, aromatase inhibitor). Estrogen mediates its functions through two specific intracellular receptors, estrogen receptor alpha and estrogen receptor beta, which act as hormone-dependent transcriptional regulators [25,26]. The estrogen receptor pathway plays a critical role in the pathophysiology of human breast cancer. Overexpression of estrogen receptor alpha is a well-established prognostic and predictive factor in breast cancer patients. The prognostic significance of estrogen receptor beta is not well defined [27-30].

Our previous study on the different series of bis-aziridinylnaphthoquinone compounds identified that they exhibit a more potent response toward the solid tumors than the circulation tumors [17]. This result was supported by other reports that there are differences in the reductive metabolism between the solid tumors and the circulation tumors [2]. Considering the importance of all the cellular reductases (e.g. NADPH cytochrome P450 reductase, cytochrome b5 reductase, [NADP]H oxidoreductase, NQO1) in response to the whole cellular reductive metabolism, these reductases are probably involved in bioactivation of AZ-1. The bioreductive drugs AZQ, mitomycin C and E09, however, have been developed to exploit the oxygen deficiency in the hypoxic fraction of solid tumors on the premise that hypoxic cells should show a greater propensity for reductive metabolism than well-oxygenated cells [2,31-33].

The major events involved in tissue homeostasis are proliferation, differentiation, and apoptosis. The processes of cell reproduction normally take place through an ordered process, generally known as the cell cycle. The tumor suppressor gene p53 is a multifunctional protein mainly responsible for maintaining genomic integrity, and it is the most frequently mutated gene in human tumors [34]. In response to DNA damage, aberrant growth signals, or chemotherapeutic drugs, p53 is stabilized and induces apoptosis and/or cell cycle arrest. While the mechanisms of p53-dependent apoptosis are not understood well, p53-dependent cycle arrest is primarily mediated by the cdk inhibitor p21 [34-36]. p21 regulates the cellular repair response to damaged DNA [37].

In the cytotoxicity results, AZ-1 induced the death effect of BC-M1 cells and MCF-7 cells in a dose-dependent and time-dependent manner in BC-M1 cells (Figs 1 and 2). From the results shown in Fig. 3, the hormone antagonist taxmoxifen and AZ-1 were more potent than paclitaxel to our local cell line BC-M1. This indicates that the BC-M1 cell is more sensitive to the hormone antagonist drug and our bioreductive compound AZ-1 than to a nonhormone antagonist such as paclitaxel. We assumed that the BC-M1 cell is an estrogen receptor-positive cell line. According to the results of Figs 4 and 5, the apoptosis phenomena were observed in BC-M1 cells induced by AZ-1 for 24 hours. We saw the apoptotic bodies increasing in direct proportion with the concentration of AZ-1 based on the sub-G_1 _area measurement and Hoechst staining (Figs 4 and 5). The apoptotic bodies were increasing slightly in 0.5 μM AZ-1, and were most abundant at a concentration of 2.0 μM. The apoptotic bodies were also observed in MCF-7 cells after treatment by AZ-1 for 24 hours (Fig. 6). For the mechanism of the apoptotic pathway, we found that p53 might be involved in the death effect of BC-M1 cells induced by the bioreductive compound AZ-1 in this study. Figure 7 shows that the expression of the p53 protein of BC-M1 cells was upregulated from 0.5 μM, was highest at 1.0 μM and downregulated at 2 μM AZ-1 for a 24-hour challenge. The response of the expression sequence in p21 protein was upregulation from 1.0 μM to 2.0 μM AZ-1 treatment to BC-M1 cells, which was later than for p53 protein. The p21 protein is the inhibitor of cdk2, the results of Fig. 8 showing that it was decreasing to 40% of the cdk2 at 2.0 μM AZ-1 and was without any disturbance to cdk2 expression in 0.5 μM AZ-1.

TIAR is a membrane of RNA recognition motif-type RNA-binding protein and also regulates the general translational arrest that accompanies environmental stress [38]. In the roles of the RNA-binding protein, TIAR involved the connections between the eukaryotic initiation factor kinase system, mRNA stability, and cellular chaperone levels [39,40]. Mutant mice lacking TIAR exhibit partial embryonic lethality and defective germ-cell maturation, implicating this protein in certain aspects of vertebrate development [41]. In DT40 cells, TIAR is also required for cell viability that involved the splicing of the exons [42]. In our results, the expression of TIAR protein was decreased with the concentrations of AZ-1 increasing in BC-M1 cells. We therefore proposed that TIAR was involved in the death effect of BC-M1 cells induced by AZ-1, which might play a role in cell viability, without direct proportion to apoptosis correlation.

According to the report of Schmidt and colleagues, p53 expression was correlated with the resistance against paclitaxel [43]. From this report, none of the tumors with p53 expression (11 patients) responded to paclitaxel. In contrast, 10 of the 22 patients without p53 expression showed an objective response. This phenomena was also supported by the report of Takara and colleagues [44], whereby the doses of paclitaxel used in BC-M1 cells are much higher than the usual IC_50 _values for most breast cancer cell lines reported in the literature (Fig. 3). This was also supported by the data from our results (Fig. 7) that the p53 protein really existed in BC-M1 cells, and with function. The report from Zhang and colleagues failed to detect the p53 protein in normal breast epithelial cells, and p53 positivity was 24% and 30% in intraductal and invasive cancer tissues, respectively [45]. The p53 protein could therefore be detected in tumor cells such as BC-M1 cells in untreated conditions, as in our results.

Caspases are a large family of cysteine proteases, most of them playing central roles in the execution of apoptosis [46]. The pro-caspase protein was in its inactive form when the cell was in the rest state and the protein expression was decreasing to convert to the active form of caspase-3 in the process of apoptosis [47]. In our result, the expression of pro-caspase protein was in inverse proportion to the concentrations of AZ-1 (Fig. 8, lane 2), and compared the enzyme activity assay of caspase-3 that was in direct proportion to AZ-1 concentration treatment to BC-M1 cells for 24 hours, as observed in Fig. 9. The apoptosis pathway of BC-M1 cells induced by AZ-1 compound was mediated by caspase-3. In this study, we also approached the apoptosis phenomena by the NMR spectra method. Some reports provided evidence that NMR spectroscopy allows the early detection (after 6 hours) of apoptosis-induced cellular changes in cells by observing signals from small and mobile molecular species, particularly the ratio of intensities of the CH_2 _and CH_3 _resonance [22,48]. Figure 10 shows that we observed that the ratio of CH_2 _and CH_3 _in BC-M1 cells induced by AZ-1 for 24 hours was also in direct proportion with the concentration of AZ-1. This is also one of the pieces of evidence to prove the apoptosis phenomena in BC-M1 cells induced by AZ-1.

## Conclusion

The apoptosis pathway in BC-M1 cells induced by various concentrations of AZ-1 was initially triggered by the activation of p53 protein and then upregulation of the p21 protein to inhibit the cyclin kinase cdk2 expression to arrest BC-M1 cell temporality and, finally, into the apoptosis process. In BC-M1 cells, the arrest and apoptosis processes, the activation of caspase-3 activity, apoptotic body formation, the ratio of CH_2 _and CH_3 _increasing and the expression of the RNA-binding protein TIAR decreasing were all involved in the death effect of BC-M1 cells induced by this bioreductive compound. From this study, AZ-1 could be used as a cocktail in combination with other anti-breast cancer drugs to cure the hypoxia tumor tissue of breast cancer patients to prevent cancer cell recurrence.

## Abbreviations

AZ-1 = 2-aziridin-1-yl-3-[(2-{2-[(3-aziridin-1-yl-1,4-dioxo-1,4-dihydronaphthalen-2-yl)thio]ethoxy}ethyl)thio]naphthoquinone; cdk = cyclin-dependent kinase; DMEM = Dulbecco's modified Eagle's medium; IC_50 _= inhibition concentration of 50% cell growth; NMR = nuclear magnetic resonance; PBS = phosphate-buffered saline; TIAR = T-cell restricted intracellular antigen-related protein.

## Competing interests

The author(s) declare that they have no competing interests.
